# Palliative care needs in patients hospitalized with heart failure (PCHF) study: rationale and design

**DOI:** 10.1002/ehf2.12027

**Published:** 2015-03-19

**Authors:** Ross T. Campbell, Colette E. Jackson, Ann Wright, Roy S. Gardner, Ian Ford, Patricia M. Davidson, Martin A. Denvir, Karen J. Hogg, Miriam J. Johnson, Mark C. Petrie, John J.V. McMurray

**Affiliations:** ^1^ BHF Glasgow Cardiovascular Research Centre University of Glasgow Glasgow Scotland UK; ^2^ Golden Jubilee National Hospital Clydebank UK; ^3^ Robertson Centre for Biostatistics University of Glasgow UK; ^4^ Johns Hopkins University Baltimore MD USA; ^5^ Edinburgh University Edinburgh UK; ^6^ Glasgow Royal Infirmary UK; ^7^ Hull York Medical School University of Hull Hull UK

**Keywords:** Heart failure, Palliative care

## Abstract

**Aims:**

The primary aim of this study is to provide data to inform the design of a randomized controlled clinical trial (RCT) of a palliative care (PC) intervention in heart failure (HF). We will identify an appropriate study population with a high prevalence of PC needs defined using quantifiable measures. We will also identify which components a specific and targeted PC intervention in HF should include and attempt to define the most relevant trial outcomes.

**Methods:**

An unselected, prospective, near‐consecutive, cohort of patients admitted to hospital with acute decompensated HF will be enrolled over a 2‐year period. All potential participants will be screened using B‐type natriuretic peptide and echocardiography, and all those enrolled will be extensively characterized in terms of their HF status, comorbidity, and PC needs. Quantitative assessment of PC needs will include evaluation of general and disease‐specific quality of life, mood, symptom burden, caregiver burden, and end of life care. Inpatient assessments will be performed and after discharge outpatient assessments will be carried out every 4 months for up to 2.5 years. Participants will be followed up for a minimum of 1 year for hospital admissions, and place and cause of death. Methods for identifying patients with HF with PC needs will be evaluated, and estimates of healthcare utilisation performed.

**Conclusion:**

By assessing the prevalence of these needs, describing how these needs change over time, and evaluating how best PC needs can be identified, we will provide the foundation for designing an RCT of a PC intervention in HF.

## Background

Heart failure (HF) is common, affecting 1–2% of the general population with the prevalence rising to over 10% in those aged over 80 years.^1^, ^2^ HF leads to a reduced life expectancy, with 5 and 10 year survival rates of 50 and 10% ,respectively, reported in epidemiological studies.^3^, ^4^ These poor survival rates have led to comparisons with cancer.^5^ Therefore, it would seem intuitive that some patients with HF may benefit from specialist palliative care (PC), given the poor survival and high symptom and care‐giver burden associated with this condition. The World Health Organization (WHO) defines PC as an approach that improves the quality of life (QOL) of patients and their families facing the problem associated with life‐threatening illness, through the prevention and relief of suffering by means of early identification and impeccable assessment and treatment of pain and other problems, physical, psychosocial, and spiritual.^6^ Contemporary HF guidelines have begun to recommend that PC be considered in patients with advanced HF refractory to optimal, or maximally tolerated, contemporary, disease‐modifying drug and device therapies, and where the resultant symptom burden is high and prognosis poor.^7^, ^8^, ^9^, ^10^ These recommendations are, however, based on relatively limited evidence and no substantial and systematic study of the actual and specific PC needs of patients with HF (and their caregivers) has been undertaken. It is possible that despite a reduced life‐expectancy, many patients with HF do not require specific or specialist PC—for example those who remain only slightly functionally limited by symptoms before a sudden death (and sudden death is relatively more common in patients with mild symptoms).^11^ However, a proportion of patients with HF almost certainly do require PC but, as yet, we do not know how large or small this group is.

Although many small studies and recent systematic reviews of these have suggested likely unmet PC needs of patients with HF,^12^, ^13^ the true prevalence of these needs is difficult to quantify for a number of reasons. As well as being small, most of the studies included highly selected patients or described cohorts with HF and a variety of other conditions. Secondly, the patients with HF were often not clearly described, and because natriuretic peptides and echocardiography were rarely used, the diagnosis of HF in some is uncertain. Furthermore, any differences in PC needs between HF with preserved ejection fraction (HF‐PEF), which accounts for half of all cases in some cohorts,^14^, ^15^ and HF with reduced ejection fraction (HF‐REF) have not been described. Most studies to date have been cross‐sectional and have not, therefore, described how PC needs change over time. Finally, many of the existing studies have used qualitative techniques, meaning their findings cannot be quantified or translated to other populations. A systematic study describing the prevalence of PC needs (and the specifics of these), using quantifiable measures, in an unselected, real‐life cohort of patients with HF, is required. As HF is a condition known to fluctuate, a single assessment, cross‐sectional study design is unlikely to identify those with a sustained need for a palliative‐intervention, and serial measurement is essential.

Whether PC is a worthwhile intervention in HF is unknown because no substantial randomized controlled clinical trial (RCT) has been performed. An RCT of PC in terminal conditions is, however, possible. One RCT of an early PC intervention in patients with lung cancer has been performed, with a suggestion that early PC can improve QOL and reduce healthcare utilisation.^16^ However, a more recent RCT of early PC in patients with advanced cancer did not show a significant improvement in the primary outcome of QOL in the early PC arm.^17^ While some of these results are encouraging, the symptoms and needs of patients with HF who are approaching the end of life may be quite different from patients with cancer, and the design of a potential RCT of PC in HF must consider this. Measures for assessing PC needs in patients with HF require further evaluation before being applied in a large RCT in HF. Another notable trial was conducted in patients deemed terminally ill as a result of a number of different illness and were housebound (33% of study participants had HF, 47% had cancer, and 21% had chronic obstructive airways disease). This trial compared usual care to usual care plus PC at home, delivered by a multi‐disciplinary team.^18^ Participants in the PC arm experienced significantly greater patient satisfaction, reduced use of medical services and healthcare costs, and were more likely to die at home than in a hospital. Although this study was well designed and informative, the cohort was mixed and the characteristics of the patients with HF were not clearly described. In particular, there was no differentiation between HF‐PEF and HF‐REF and no description of HF treatment. More recently, a single centre, RCT of PC in HF, randomized 72 outpatients with HF‐REF NYHA class III/IV to receive either early PC and HF care together or standard care.^19^ Assessments of QOL and symptoms were made at 1, 3 and 6 months and patients were followed up for HF hospitalisations for 6 months. The PC arm experienced greater improvements in QOL and had fewer HF hospitalisations. These results are encouraging, however, the numbers of patients were small and patients with HF‐PEF were not represented. A large RCT of PC in HF is needed to build upon these preliminary findings. Before such a trial can be designed, suitable outcome measures and tools for identifying patients with PC needs must be assessed in a real‐life population of patients with HF.

### Study aims

Ultimately, the goal of this study is to provide the foundation for an RCT of a PC intervention in HF. This will include characterisation of an appropriate study population, identifying the components of a specific and targeted PC intervention, and defining potential trial outcomes. To achieve this goal, we will address the following specific points:

1. Describe and quantify the supportive and PC needs of a cohort of patients hospitalized with HF (and the needs of their caregivers). Specifically, describe and quantify general and disease‐specific QOL, symptom burden, caregiver burden, mood, performance status, and preferences for end of life (EOL) care of these patients.
2. Evaluate whether estimated poor prognosis or recognized assessment tools used in other populations (e.g. patients with cancer) can identify patients with hospitalized HF who have PC needs.
3. Describe the healthcare utilisation of patients hospitalized with HF, and describe any differences between those with and without PC needs.
4. Using the findings from items 1–3, design an RCT of a PC intervention for HF. In particular, we will identify an appropriate population with a high PC need (and describe how to identify this need), define the components of an HF‐specific and targeted PC intervention, and suggest potential outcome measures for such a trial.

## Rationale and study design

### Describing the supportive and palliative care needs of patients with heart failure

Although there have been descriptions of unmet supportive and PC needs of patients with HF, these needs have not been described using reproducible and quantifiable measures in a ‘real world’ HF population. Any such description should take into account the WHO definition of PC,^6^ and therefore not only make an assessment of EOL care needs, but also the QOL of patients and their caregivers, mood and symptom burden.

### Quality of life

It is unclear which QOL tool is best in patients with HF, although a combination of a HF specific and a generic questionnaire may be optimal.^20^ One of the most commonly used HF specific questionnaires is the Kansas City Cardiomyopathy Questionnaire (KCCQ).^21^ This has been widely used in a number of HF studies and has been well validated.^22^, ^23^, ^24^, ^25^ A variety of generic QOL assessment tools are available and one of the most widely used is the SF‐36,^26^ which has been validated in a variety of populations including HF.^27^, ^28^ This has been shortened to a 12 question format, while retaining validity,^29^ in the form of the Short Form 12 (SF‐12).^29^, ^30^

Heart failure not only affects patients' QOL, but also that of their caregivers.^31^ Caregiver QOL can be readily assessed using a generic tool, such as SF‐12, but assessing ‘caregiver burden’ within a family as a result of HF can also be assessed using the Zarit Burden Interview.^32^ This is the most widely used and validated caregiver assessment tool.^33^ This tool also includes an assessment of financial strain placed on the caregiver. This is an important question, as patients within the last 6 months of life are potentially entitled to financial support in some countries.

### Symptoms

Assessing on‐going symptoms should also form part of an assessment of potential PC needs in HF patients nearing end‐of‐life. HF trials tend to focus on the symptoms of dyspnoea, fatigue, and oedema. However, it has recently been shown that patients with HF can develop a multitude of other symptoms including pain, anxiety, low mood, constipation, anorexia, nausea, insomnia, and persistent cough.^34^, ^35^ There are recognized tools to help make an objective measurement of symptom burden but these have not been extensively evaluated in HF (although they have been in other diseases). The Edmonton Symptom Assessment System (ESAS)^36^ has been validated in cancer^37^, ^38^ and has previously been used in a study of patients with HF.^39^ Scrutiny of the content of the ESAS and this prior study suggest that it is able to quantify many of the symptoms experienced in HF.

### Mood assessment

The WHO definition of PC states that PC should identify and treat psychosocial and physical problems. Therefore, any assessment of PC needs should detail how QOL, symptom burden, and end‐of‐life care potentially affect a patient's mood. Indeed, in one RCT of PC use in lung cancer, mood assessment was used as an outcome measure. Depression, which is common in HF,^40^ can affect QOL^41^ and is associated with higher morbidity and mortality.^42^, ^43^, ^44^ A validated screening questionnaire for depression is the Hospital Anxiety and Depression Scale (HADS).^45^ This has been used in a variety of populations,^46^ and is validated in HF.^47^

### End of life care

Most patients with HF die from cardiovascular (CV) causes, the majority of which are from either worsening HF or sudden cardiac death.^48^ There is some evidence that patients with more severe symptoms of HF are more likely to die from worsening HF, whereas less symptomatic patients are more likely to suffer sudden cardiac death.^11^, ^49^ Not every patient with HF who dies will have unmet palliative needs, e.g. those who die from sudden unexpected cardiac death with little functional limitation. However, patients with progressive HF may benefit from palliative intervention, and how these individuals are best identified is currently unknown.

The majority of HF patients currently die in hospital. Place of death was recorded in the Assessment of Treatment with Lisinopril and Survival (ATLAS) trial with more than 50% of patients dying in hospital. Among those who died out of hospital, the mode of death was most likely to be sudden.^50^ These findings were similar to those of the Sudden Cardiac Death in Heart Failure Trial (SCD‐HeFT) where 58% of patients died in hospital, 29% died at home, and 7% in an extended care facility.^51^ Although these clinical trials are informative, they included selected populations and are not representative of ‘real world’ patients with HF. A recent analysis of data from death certificates from England and Wales reported that over 60% of patients dying from HF died in hospital and <20% died at home.^52^ However, HF is under‐reported on death certificates,^53^, ^54^ and this finding may not reflect the true experience of patients with HF. Preferred place of death when recovery seemed unlikely was described in a study of 80 patients hospitalized with HF which reported that 50% wished to be cared for at home, 40% wished to remain in hospital, and 10% were unsure.^55^ Data comparing preferred place of death to actual place of death are lacking in an unselected cohort of patients with HF.

Patient preference for EOL care has been identified as a priority for research into PC need in HF.^10^, ^56^ Previous studies suggest that patients often change their mind about preferred place of death, and there is also poor agreement with their caregiver on this issue.^57^ Patients also change their mind about resuscitation status.^58^, ^59^ A recent study showed that HF patients were willing to discuss EOL issues and that patients were willing to trade QOL for length of life,^60^ which is contrary to previous studies.^61^, ^62^ Although these studies are informative, they are based on selected cohorts of patients.

Any EOL assessment should not only assess preference for and actual place of death, but also the patient and caregiver experience of dying, wherever that occurs. The Views of Informal Carers for the Evaluation of Services (VOICES) postal questionnaire has been designed to evaluate relative's experience of EOL care of the patients in the last few months of life.^63^ This questionnaire has been validated, and a recent review by the United Kingdom's Department of Health has identified this as an appropriate measurement tool and the tool of choice in a survey of EOL care.^64^

### Identification of patients with palliative care needs

Before an RCT of PC use in HF can be planned, there is a need to further explore how patients with PC needs can be identified and which require the additional services of a specialist PC service. HF Guidelines suggest using the following factors to identify patients with HF and PC needs: frequent admission to hospital with decompensated HF; weight loss and cachexia; the need for frequent or on‐going intravenous therapy; chronic poor QOL with New York Heart Association (NYHA) class IV symptoms; and a clinical judgement that the patient is close to the EOL.^7^, ^10^ However, predicting prognosis is notoriously difficult and is recognized as a barrier to PC referral in HF.^65^ A number of prognostic models have been described from various HF cohorts.^66^ Unfortunately, most models were developed in chronic ambulatory populations (as opposed to acutely hospitalized patients) and many were based on patients not receiving contemporary pharmacotherapy, or did not include important prognostic factors such as renal function, B‐type natriuretic peptide (BNP)^67^ (or NT pro BNP) or troponin.^68^, ^69^ Use of prognostic models has been suggested as a way of identifying HF patients who are approaching EOL.^70^ However, while these models may predict death, there is no evidence to suggest that prognostic models correlate well with PC needs.^71^ This question requires further exploration.^72^

One approach to try and specifically identify and assess the PC needs of HF patients is to use tools currently in development for cancer patients, acknowledging that these require assessment in patients with HF. The Needs Assessment Tool (progressive disease – cancer) (NAT‐PD‐C)^73^ has been designed specifically to assess PC needs in cancer patients. It was designed based upon a literature review of needs of patients and their caregivers. This assessment is made on a single page and completed by the patient's healthcare professional. The NAT‐PD‐C has been validated in cancer patients^74^, ^75^ and has been adapted for use in HF with the creation of the Needs Assessment Tool Progressive Disease Heart Failure (NAT‐PD‐HF), with reliability testing and construct validation.^76^ However, this tool has yet to be evaluated in a substantial cohort of patients with HF, and its value in identifying PC needs in patients with HF is as yet unconfirmed. Therefore, we will carry out these evaluations of the NAT‐PD‐HF.

Performance status has been used by PC clinicians in both clinical practice and research as an indication for the likely need for PC services.^77^, ^78^, ^79^ The Karnofksy Performance Scale (KPS)^80^ is regarded by many as the gold standard tool for use in cancer patients.^77^, ^78^ This instrument has been simplified and validated in the form of the Australia‐Modified Karnofksy Performance Scale (AKPS).^81^ The AKPS has been developed for use in cancer, and review of it suggests that it should also provide a suitable assessment of performance status in patients with HF.

**Figure 1:**
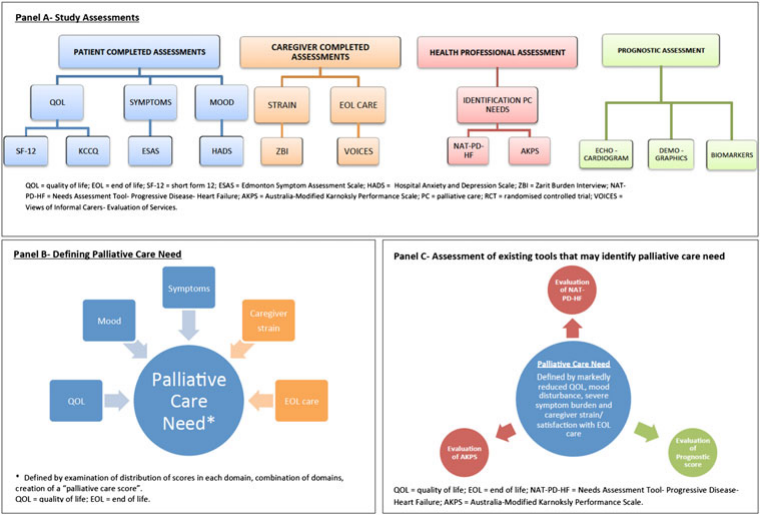
Study design.

An overview of the study design and the outcome measures used are illustrated in *Figure* 1, Panel A.

### Study protocol

**Figure 2:**
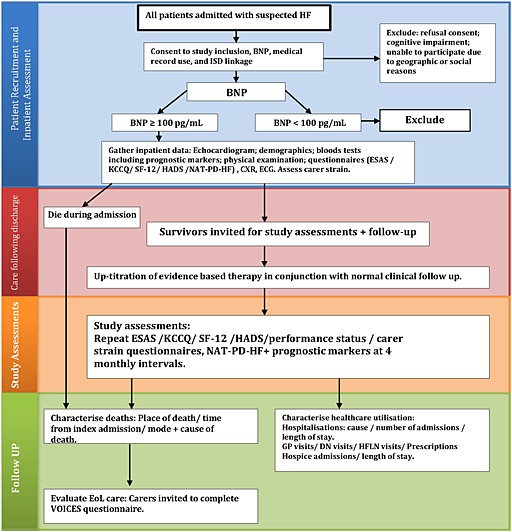
Study protocol.

This will be a 2‐year prospective observational study of near‐consecutive patients admitted to hospital with HF. An outline is described in *Figure* 2. Patients will be extensively characterized during their inpatient stay by collecting echocardiographic (*Table* 1), demographic, and physiological data, as well as a detailed past medical history. Patient symptom burden, mood, and QOL will be assessed during the index admission and repeatedly during follow up. The burden on caregivers will also be assessed. Patient preference for place of death (and actual place of death, if death occurs), as well as resuscitation preference, will be recorded. Health care utilisation will be evaluated.

**Table 1:** Echocardiographic protocol and data

	Protocol		Measurement
Window	Doppler	2D/M‐mode	
Parasternal			
Long axis	MV & AV colour flow	IVSd, LVEDD, LVPWd	LV end diastolic dimension (cm/m^2^)
		IVSs, LVESD, LVPWs, LVOT, LA	LV end systolic dimension (cm/m^2^)
RV inflow	TV CW + colour flow		
Short axis			
Base	AV, TV & PV colour flow		
MV	MV colour flow		
Papillary muscle		2D endocardial & epicardial area	LV mass index (g/m^2^)
apex			
Apical			
4 chamber	MV annulus TDI + LV inflow PW	LV volume diastole + systole, LAA	LV EF (%)
	MV colour flow, TV colour flow		LV diastolic volume (ml/m^2^)
			LV systolic volume (ml/m^2^)
			LV stroke volume (ml)
			Cardiac output (L/min)
			LV diastolic parameters (E, E/e’, IVRT, E/A)
			Left atrial volume (ml/m^2^)
			Valve assessment of structure and function
2 chamber	MV colour flow	LV volume diastole + systole, LAA	LV EF (%)
			Left atrial volume (ml/m^2^)
			LV diastolic volume (ml/m^2^)
			LV systolic volume (ml/m^2^)
5 chamber	AV CW + PW + IVRT, AV colour flow		
Long axis	MV colour flow		
RV		TAPSE, RAA	TAPSE
			Right atrial area (mm^2^)
Subcostal			
4 chamber			
IVC & hepatic veins		IVC diameter	RVSP

AV, aortic valve; CW, continuous wave; E, early diastolic filling; e’, early lengthening velocity; IVC, inferior vena cava; IVRT, isovolumic relaxation time; IVSd, intraventricular septal diastole; IVSs, intraventricular septum systole; LVEDd, left ventricular end‐diastolic dimension; LVESD, left ventricular end‐systolic dimension; LVPWd, left ventricular posterior wall diastole; LVEDs, left ventricle posterior wall systole; EF, ejection fraction; LAA, left atrial area; LV, left ventricle; MV, mitral valve; PV, pulmonary valve; PW, pulsed wave; RV, right ventricle; RVSP, right ventricular systolic pressure; TAPSE, tricuspid annular plane systolic excursion.

## Patient recruitment

Near consecutive patients admitted to the Western Infirmary in Glasgow with suspected HF will be screened for inclusion in the study. HF will be defined according to the European Society of Cardiology (ESC) guidelines.^10^ The Western Infirmary acts as a community hospital for the North and West of the city, serving a population of about 250 000. All patients admitted with a primary diagnosis of clinically suspected HF will be approached and asked for permission to access their medical records and to link their record through National Health Service Scotland Information Services Division (ISD), allowing identification and cause of hospital readmission and death (including place of death). Plasma BNP will be measured to aid the diagnosis of HF (and will provide prognostic information). A finger prick (12 μL) sample of blood will be analysed for BNP using a validated, point of care, capillary blood sample analysis (Alere HeartCheck System). Those with a BNP <100 pg/mL will be excluded.^10^ In addition to elevated BNP, patients must meet the ESC echocardiographic criteria for the diagnosis of HF.^10^, ^82^ Patients with a confirmed diagnosis of HF will be invited to participate in the study, and a further sample of blood and urine will be taken and stored for later batched analysis of biomarkers, which have prognostic importance in HF. In a previous study collecting data on near‐consecutive HF admissions, we recruited almost 350 patients at the Western Infirmary over a 2‐year period.^83^ and we anticipate similar recruitment in this study. Based on our earlier study, we anticipate that approximately 30% of patients will die in the first year of follow‐up and approximately 15% per year thereafter.

Inclusion and exclusion criteria are detailed in *Table* 2.

**Table 2:** Inclusion/ exclusion criteria

Inclusion criteria	Exclusion criteria
• Admitted to hospital with a primary diagnosis of acute decompensated HF	• Refusal to participate
• Age ≥18 years	• Unable to provide informed consent/complete study assessments
• Fulfilling the ESC diagnostic criteria for the diagnosis of HF	○ Confusion/dementia
• HF‐REF, HF‐PEF and valvular HF will be included	○ Learning difficulties
	○ Unable to read or write English language
	○ Moribund
	• Readmission
	• Geographical reasons, not from catchment area
	• Isolated cor pulmonale
	• Acute coronary syndrome complicated by pulmonary oedema

ESC, European Society of Cardiology; HF, heart failure; HF‐REF, heart failure with reduced ejection fraction; HF‐PEF, heart failure with preserved ejection fraction.

## Patient assessment

Detailed clinical data and data used in validated models of mortality prediction in HF will be gathered during the index hospitalisation.^66^ A full echocardiographic examination will be carried out according to the European Association of Echocardiography guidelines,^84^ and assessment of known prognostic variables will be recorded (*Table* 1). Left ventricular ejection fraction will be measured using Simpson's biplane method.^85^ Before discharge from hospital, patients will complete the KCCQ and SF‐12 questionnaires to assess QOL and the ESAS questionnaire to assess their current symptom burden. Mood assessment will be made with the HADS questionnaire. Patients' caregivers will be invited to complete the Zarit Burden Interview to assess caregiver burden. Performance status will be evaluated using the AKPS. The NAT‐PD‐HF will be used to assess the palliative needs of the patient. One of the investigators will also ask about preferred place of death and resuscitation preference, in a sensitive way, in patients thought to be near to death and in those who exhibit significant deterioration during follow‐up. Patients will be asked to consider their preference for place of care, specifically, patients will be asked ‘If your health was to deteriorate in the future, such that you required other people to care for you, where would you prefer that care to take place?’ Patients will then be given the following options to choose from, after explaining this is a hypothetical discussion: in their own home; a nursing or care home; hospital; hospice; or undecided. Patients will then be asked to consider their preferred place of care for EOL treatment, specifically, patients will be asked ‘If you were to think about the last few days of hours of life, would you have a strong opinion or preference for where that care took place?’ Patients will then be given the following options to choose from, after explaining this is a hypothetical discussion: in their own home; a nursing or care home; hospital; hospice; or undecided. Finally, patients will be asked, after an explanation of what resuscitation is, to consider their preference for resuscitation. Specifically, patients will be asked ‘Do you have a strong opinion or preference to be resuscitated or not to be resuscitated in the event of a cardiac arrest’. Patient will be asked to pick an option from ‘for active resuscitation, not for resuscitation, or undecided’. The researcher will only ask these questions where it is felt to be appropriate, in patients approaching the EOL, and only after making explicitly clear that the questions relate to research and are not for clinical purposes. While we recognize the limitations in predicting likely death in HF, our Ethics Committee does not think it appropriate that we approach all patients about this question.

## Study assessments

Following discharge, patients will be reviewed at an outpatient clinic by a cardiologist, a Heart Failure Liaison Nurse (HFLN), or both, where evidence‐based therapy will be optimized in accordance with ESC guidelines.^10^ Patients will be invited to attend for study assessments at 4‐monthly intervals following discharge for a maximum follow‐up period of 2.5 years. At these visits, KCCQ, SF‐12, HADS, and ESAS questionnaires will be completed, to detail any potential change in QOL, mood, and symptom burden over time. EOL preferences will also be re‐evaluated (as appropriate), including preferred place of care/death and resuscitation preference. At these study assessments, prognostic markers will be updated and the NAT‐PD‐HF will be reassessed. To ensure a complete follow‐up as possible, we will contact those unable to attend study visits by telephone and offer home visits where possible.

## Follow‐up

All patients who consent will be ‘flagged’ using ISD linkage, ensuring complete follow‐up (all will be followed up for a minimum of 12 months). PC could potentially alter Healthcare utilisation.^16^ Therefore, the number of hospital admissions, length of stay, cause of admission, general practitioner (GP) visits, district nurse (DN) visits, hospice admissions, HFLN visits, and prescription costs will be recorded. These data including date, cause and location of death will be available from ISD and GP records.

Relatives of deceased patients will be asked to complete the VOICES EOL postal questionnaire. Relatives will be written to and given the opportunity to opt out prior to the questionnaire being posted.

## Data handling and statistical analysis

All data will be managed and analysed by the Robertson Centre for Biostatistics (University of Glasgow) and the Data and Biostatistics Centre of the UK Clinical Research Collaboration Glasgow Clinical Trials Unit (CTU). Baseline and follow‐up data (including patient and carer QOL) will be entered into a case report form and then into the study database by experienced data entry staff. All data will be stored, managed, and analysed according to CTU standard operating procedures that comply with appropriate legal and regulatory requirements. We will define patients in need of PC as those reporting a substantial reduction in QOL, marked mood disturbance, or severe symptoms (as measured by NYHA class, KCCQ, and ESAS), especially if there is associated caregiver strain (Figure 1, Panel B). We will test whether the NAT‐PD‐HF and AKPS tools (and a prognostic score derived from standard clinical assessments) identify these patients. Much of the analysis will be descriptive, using different scores and combinations of scores from the various patient‐reported outcomes to define a need for PC (Figure 1, Panel C). We will also assess temporal variations in QOL, patient symptom burden, and PC needs for the cohort as a whole and for different patient sub‐groups. The relationship between the need for PC as identified by the NAT‐PD‐HF and other clinical and QOL instruments will be analysed using logistic regression models. The relationship between baseline characteristics, QOL data, and mortality will be assessed using logistic regression and time to event analyses. The additional value of repeated QOL assessments will be evaluated using time varying covariate Cox models.

## Ethical considerations

This study will be conducted according to the principles outlined in the Declaration of Helsinki.^86^ The study protocol has been approved by the West of Scotland Research Ethics Committee. All participants will be given over 24 h to read the patient information letter and consider if they wish to participate before provide written consent. Patient burden and load have been considered, and patient reported outcome measures have been chosen to limit the burden placed on participants. Burden of follow‐up study visits has been reduced by offering participants home visits or providing door‐to‐door transport. Our local Ethics Committee has asked that we only ask preferred place of death in patients thought to be near to death and in those who exhibit significant deterioration during follow‐up. While we recognize the limitations in predicting likely death in HF, our Ethics Committee does not think it appropriate that we approach all patients about this question.

## Discussion

Performing research in people with serious illness is difficult, with poor recruitment, and high rates of participant dropout common,^87^ perhaps explaining why few studies of PC in HF have been longitudinal, quantitative, or performed in large cohorts. Barnes *et al.* described their experience of recruiting a large cohort of elderly patients with HF into a longitudinal study,^88^ and reported such problems. They experienced difficulties with recruitment and retention of elderly patients, with only 30% of patients approached agreeing to participate. One of the main challenges they had was the reliance on a gate‐keeper to recruit participants, in their case the GP (but in other similar studies often hospital specialists).^89^ They found some GPs to be over‐restrictive in recruitment, excluding potential participants for reasons outside the protocol‐specified exclusion criteria, particularly elderly patients living in care facilities. Other GPs did not utilize the exclusion criteria, and therefore ineligible patients were also approached. These factors can introduce selection bias, reducing the generalisability of results. One of the key strengths of our study is the reduction in selection bias by approaching a consecutive, and thus recruiting a relatively unselected, cohort of hospitalized patients. We will reduce the aforementioned issues by not relying on a gate‐keeper physician to recruit participants, as a member of the study team will screen all admissions for potential participants.

Another potential issue highlighted by Barnes *et al.* was the identification of patients with HF. They searched patient databases for the diagnostic code ‘heart failure’ and combining this with a search for patients on a regular loop diuretic. Although this screening method is efficient and has the advantage of high sensitivity, specificity is likely low. The diagnosis of HF is difficult, especially HF‐PEF,^90^ and usually requires the combination of clinical signs and symptoms, natriuretic peptides, and echocardiography.^10^ This highlights a further strength of our study as all potential participants will be screened using natriuretic peptides and echocardiography, making the cohort one of the best described contemporary HF populations in the field of palliative care.

Participation burden has also been identified as a potential issue affecting retention of participants in any longitudinal study of patients with HF,^88^ with some outcome measures, such as filling in questionnaires or attending study assessments, becoming more challenging for participants to complete as their condition progresses and their general health deteriorates. This has been considered, and measures and study design chosen to reduce participant burden as much as possible. This is one of the main aims of this study, to pilot the use of many of the outcome measures over time in a frail population, to help inform the design of a large RCT of early PC intervention in patients with HF.

The main weakness of our study is the exclusion criteria. We will exclude patients with cognitive impairment such that they are unable to provide informed consent or complete study assessments, or those unable to complete study assessments because of language or communication difficulties. Although these criteria are necessary, we will potentially exclude not only a common group of patients,^91^ but those with some of the greatest needs.^92^ Targeting inpatients could also result in some patients being too unwell to participate; however, we feel the strength of recruiting an unselected hospital cohort outweighs this potential weakness.

## Conclusion

This study will be one of the largest assessing PC needs in a largely unselected cohort of patients admitted to hospital with HF, and will be one of the best‐described contemporary HF cohorts. We will assess the prevalence of PC needs and describe how these needs change over time, and assess whether those with PC needs can be identified. In doing, so we will provide the foundation for designing a large RCT of an early PC intervention in HF.

## Conflict of interest

None declared.

## Funding

This project is supported by a project grant from the British Heart Foundation; Grant number PG/13/17/30050.
